# High malaria parasitemia among outpatient febrile children in low endemic area, East-Central Tanzania in 2013

**DOI:** 10.1186/s13104-020-05092-4

**Published:** 2020-05-24

**Authors:** Beatrice Chipwaza, Robert D. Sumaye

**Affiliations:** 1 St Francis University College of Health and Allied Sciences, P.O. Box 175, Ifakara, Tanzania; 2 Ifakara Health Institute, Ifakara, P.O. Box 53, Ifakara, Tanzania

**Keywords:** Malaria, Parasite density, Children, Kilosa district, Tanzania

## Abstract

**Objective:**

This study investigated the prevalence and distribution patterns of malaria in Kilosa district as part of non-malaria causes of febrile illnesses in children study. We enrolled febrile patients aged 2–13 years presenting at the outpatient department during the rainy and dry seasons, in 2013. For each participant, we tested for malaria parasites and identified parasite species using microscopy. We then calculated parasite density and estimated geometric mean parasite density.

**Results:**

The overall malaria prevalence in febrile children was 23.7% (n = 609). *Plasmodium falciparum* accounted for 98.6% of malaria positives. There was a heterogeneous distribution of malaria cases among the 17 wards constituting the catchment area. A high proportion (69.4%, n = 144) of malaria positive individuals had high parasite densities. Individuals who were enrolled in the rainy season had higher geometric mean parasite density (15415.1 parasites/µl, 95% CI 10735.3–22134.9) compared to the dry season (6115.3 parasites/µl, 95% CI 4237.8–8824.6). The relatively high malaria prevalence recorded in Kilosa, an area considered low endemicity, calls for concerted effort in documenting malaria burden at fine geographical scales and tailor preventive and control strategies that target hotspots of high malaria transmission.

## Introduction

Despite the continued efforts geared to reduce malaria case incidence and death rates, malaria remains a major disease, especially in children. The 2018 World malaria report estimates about 219 million malaria cases occurred in 2017 which is an increase of about 3.5 million cases compared to 2016 [1], and accounts for about 435,000 annual mortalities. Importantly, the majority of these cases occur in Sub-Saharan Africa where after every 2 min a child dies from malaria. In Tanzania, malaria is endemic in most parts of the country and over 90% of the population lives in areas where malaria is endemic. Malaria accounts for over 30% of the national disease burden and is the major cause of hospital attendance, admission and death where it accounts for about 16 million clinical cases and over 100,000 deaths annually [2].

Diverse malaria control efforts have been implemented for several years in malaria-endemic areas. However, we have been observing rises and falls in malaria cases and deaths globally and Tanzania. The 2015 World malaria report shows a global decrease from about 262 million cases in 2000 to 213 million in 2015 and the decline in malaria deaths by 48% [3]. Similarly in Tanzania, the overall prevalence of malaria among children age 6–59 months has declined from 17.7% in 2008 to 9.2% in 2012 [4] and 7% in 2017 [5].

There are variations in the malaria burden between regions and districts of Tanzania. This includes differences in entomological inoculation rates, vector composition, and transmission intensities at districts level or even within villages [6–9]. Malaria prevalence ranges from as low as < 1% in Zanzibar archipelago to about 27% in the Western zone [10] with a higher burden in rural compared to urban areas [5]. Based on these variations, regular updates of malaria morbidities in different settings are invaluable. Understanding the status of malaria prevalence in different localities will allow for better planning of control strategies. This study aimed to investigate malaria prevalence among children who attend the outpatient department of Kilosa district hospital, as part of a study on non-malaria causes of febrile illnesses.

## Main text

### Methods

#### Study site and population

This study was conducted at Kilosa district hospital in the Morogoro region, Tanzania for 6 months covering both the rainy and dry seasons in 2013, as part of a large study on non-malaria causes of febrile illnesses in children [11, 12]. Kilosa district experiences the main rainy season between March–May and dry season from June–October. The main economic activities are crop production and livestock keeping. The district is divided into 38 wards and 164 villages [13]. In 2012, the population of Kilosa district was 438,175 people, in which the number of children aged 2–13 years was 146,891 [14]. The participants enrolled in the study came from 17 out of 38 wards, with the inhabitants in this catchment area accounting for about half of the district population. There were 71 healthcare facilities, of which 3 are hospitals, 7 health centers, and 61 dispensaries. The district hospital serves as a referral hospital for primary healthcare facilities (dispensaries and health centers) but also patients who directly seek treatment at the hospital. Malaria has been accounting for more than half (55.5%) of outpatient visits and is the leading cause of deaths in children < 5 years. In the year 2012 over one-fifth of deaths were caused by malaria [15].

#### Inclusion criteria, participants enrolment and procedures

The study participants were patients presenting at the outpatient department (OPD), aged 2–13 years and with axillary body temperature ≥ 37.5 °C. For 6 months each working day (excluding weekends) the study team enrolled 5 participants who met inclusion criteria, agreed to participate and the parent/guardian provided written informed consent. For each participating child, a standardized questionnaire was administered to the parent/guardian to capture demographic and clinical information. The management of patients was performed according to the local standard of care based on world health organization (WHO) and Ministry of Health guidelines [16, 17], and the same information was also recorded in the standardized questionnaire.

#### Diagnosis of malaria

A thick and thin blood smears were prepared from a finger prick blood sample and stained with 3% Giemsa solution, and examined under a light microscope with oil immersion. These were used for the identification and quantification of the malaria parasite respectively. A smear was considered negative if no parasites were seen after viewing at 100× high-power fields. For quality assurance, each slide was read independently by a second microscopist and any discrepancies were resolved by a third microscopist. The parasite density was determined according to the number of parasites per 200 white blood cells (WBC) counts, assuming a total WBC count of 8000/μL [18].

#### Parasitaemia case definitions

Parasitaemia was categorized as low (< 1000 parasites/µl blood), moderate (1000–4999 parasites/µl blood), high (5000–99,999 parasites/µl blood), and hyperparasitaemia (≥ 100,000 parasites/µl blood) [19–21]. Fever was classified as mild if the temperature was 37.5–38.3 °C, moderate at 38.4–39.4 °C and high at 39.5 °C.

#### Data management and analysis

Data were entered into an Access database, verified and cleaned to ensure that clinical data and laboratory findings are matched. The statistical analyses were performed using STATA software (version 13; Stata Corp., TX USA). The geometrical mean of parasite density (GMPD) was calculated as the antilogarithm of the arithmetic mean of the base 10 logarithms of 10 plus the parasite density. Pearson’s Chi Square test was used to determine the association between categorical variables. An alpha level of 0.05 was used for all tests of statistical significance.

### Results

#### Demographic characteristics

A total of 609 participants were enrolled, of these 51.2% (n = 312) were males and 48.8% (n = 297) females. Among the 609 participants, 354 (58.1%) were children ˂ 5 years and 255 (41.9%) had ≥ 5 years. About 50% (310) of patients were enrolled during the dry season while 299 (49.1%) were enrolled in the rainy season. During the time of enrollment, the majority of the participants (78%) had a mild fever. Overall 177 (29.9%; n = 609) had prior treatment and 154 (25.3%; n = 609) of those with prior treatment were treated with antimalaria drugs. Only 29 (4.8%; n = 609) of those treated with antimalaria drugs were later diagnosed with malaria.

#### Malaria prevalence and distribution

**Fig. 1:**
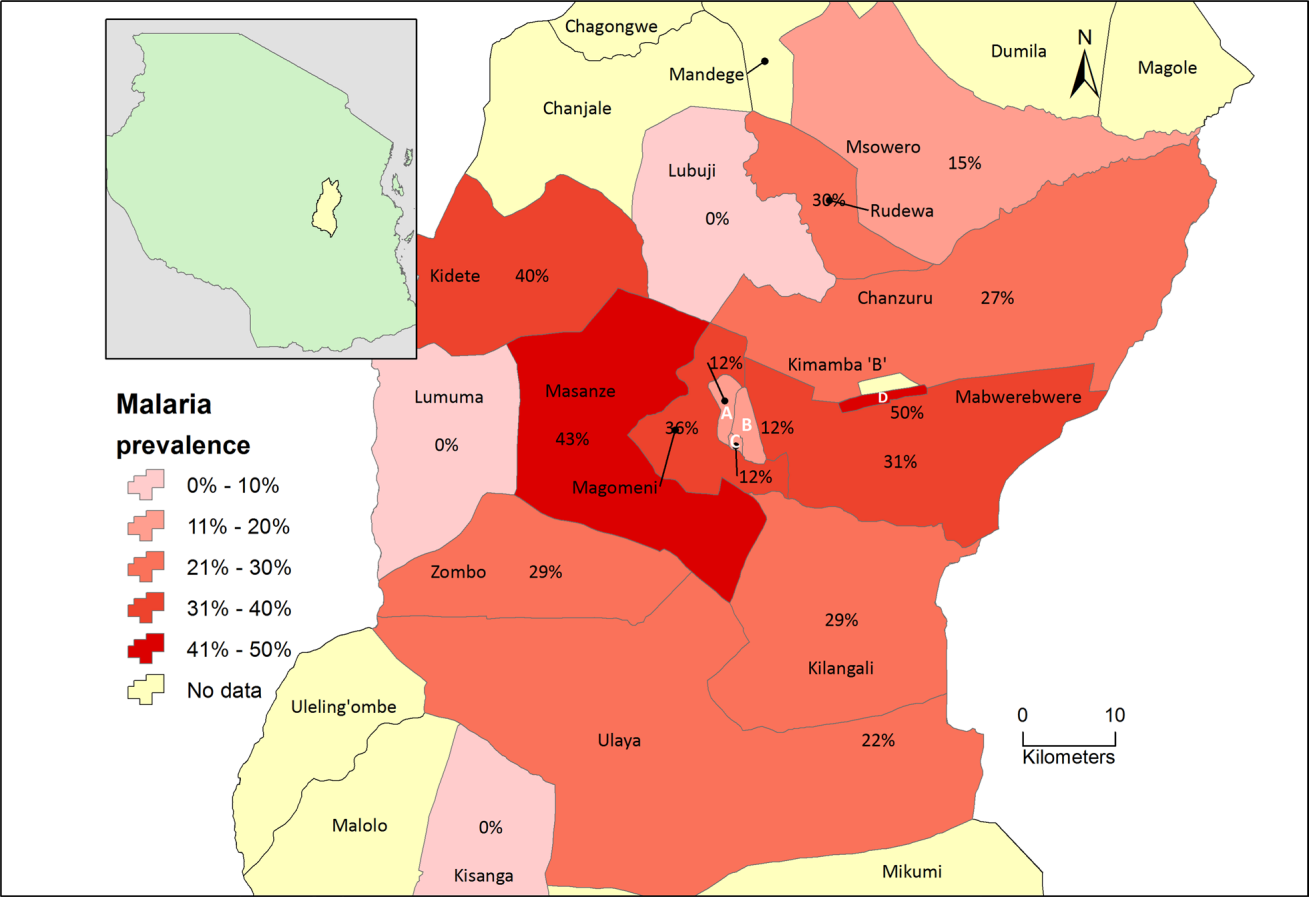
The geographical distribution of malaria prevalence in Kilosa district by wards. NB: A = Kasiki; B = Mkwatani; C = Mbumi; D = Kimamba A

The overall malaria prevalence was 23.7% (n = 609) and *Plasmodium falciparum* accounted for 98.6%. Two patients (1.4%) had a mixed infection with *P. falciparum and P. malariae.* There was no difference in malaria prevalence by season, sex and age groups, Table 1. There was a heterogeneous distribution of malaria within the wards. Out of the 144 malaria positive patients, the highest prevalence (31–50%) was recorded from individuals living in six wards i.e. Kimamba A, Masanze, Kidete, Magomeni, Mabwerebwere and Rudewa as indicated in Fig. 1, while the prevalence was 21–30% in individuals from six wards i.e. Mabwerebwere, Rudewa, Zombo, Kilangali, Chanzuru, and Ulaya.

**Table 1:** Malaria prevalence and geometric mean parasite density (GMPD) stratified by sex, age group, and season

Variable	Category	Malaria prevalence (n)	GMPD (per µl)	95% CI	*P* value
Overall	N/A	23.65% (609)	9834.62	7539.97–12827.6	
Sex	Male	26.28% (312)	8618.75	5902.98–12583.94	0.86
	Female	20.88% (297)	11710.06	8101.48–16925.98	
Age group	< 5 years	24.29% (354)	9779.53	6777.71–14110.83	0.52
	≥ 5 years	22.75% (255)	9916.86	6734.94–14602.11	
Season	Rainy	23.87% (310)	15415.1	10735.33–22134.88	0.0002
	Dry	23.41% (299)	6115.26	4237.75–8824.59

#### Malaria parasitaemia and parasite density

A high proportion (69.4%, n = 144) of malaria positive individuals had high parasite densities whereas only 2.1% had hyperparasitaemia, Table 2. The overall geometric mean parasite density was 9834.6 parasites per µl of blood. The GMPD was significantly higher during the rainy season (15415.1 parasites/µl, 95% CI 10735.3–22134.9) than the dry season (6115.3 parasites/µl, 95% CI 4237.8–8824.6). There was no difference in GMPD by sex and age groups, Table 1.

**Table 2:** Malaria parasite density among malaria positive individuals stratified by season, sex, age group, and fever category

Variable	Level	Parasite density			
		Low % (n)	Moderate % (n)	High % (n)	Hyper % (n)
Overall	na	11.8% (17)	16.7% (24)	69.4% (100)	2.1% (3)
Season	Dry	6.9% (10)	11.1% (16)	30.6% (44)	0.0% (0)
	Rainy	4.9% (7)	5.6% (8)	38.9% (56)	2.1% (3)
Sex	Female	3.5% (5)	6.3% (9)	32.6% (47)	0.7% (1)
	Male	8.3% (12)	10.4% (15)	36.8% (53)	1.4% (2)
Age group	< 5 years	7.6% (11)	10.4% (15)	39.6% (57)	2.1% (3)
	≥ 5 years	4.2% (6)	6.3% (9)	29.9% (43)	0.0% (0)
Fever category	Mild	6.9% (10)	10.4% (15)	45.8% (66)	1.4% (2)
	Moderate	4.9% (7)	6.3% (9)	19.4% (28)	0.7% (1)
	High	0.0% (0)	0.0% (0)	4.2% (6)	0.0% (0)

## Discussion

We report here high malaria parasitaemia which might imply our study population is sitting at a transmission hotspot in a low prevalence designated Morogoro region. There are relatively few studies in Tanzania reporting parasite density, a report in Muheza [22] had comparable findings with ours, whereas low density among children is reported in other places [23, 24]. In other countries also reports indicate low parasitaemia in a high proportion of children [25, 26], however, the high parasite densities recorded during the rainy seasons were comparable with findings from Nigeria [27].

The overall malaria prevalence reported here was higher compared to the data from the 2011–2012 malaria indicator survey (13%) for the Morogoro region [28], and *P. falciparum* accounted for 99% of cases. It is however comparable to data from Kigoma (26%) and Lindi (26%) regions in the Western and South-eastern zones respectively, the areas considered having the highest prevalence in Tanzania [28]. In the same catchment area, a 2015 study involving community sampling (random sampling of household members) irrespective of age and fever status reported 17.5% of malaria prevalence that was still high [29]. These observations suggest the presence of high transmission hotspots despite a sustained overall decline in malaria prevalence in children over time in the Morogoro region. Reports from other parts of Tanzania have revealed a declining prevalence of malaria in children including the Mwanza region (9.5%), Muheza (16.4%) and Korogwe (8.3%) districts in the year 2014 [30–32].

The prevalence was not different between age groups, sex and seasons. Similar observations were reported in the same district where malaria infection was associated with the age groups of 1–10 years and 11–20 years [29]. Our findings are also in agreement with a systematic review indicating an evenly distributed clinical malaria incidence across the first 10 years of life for all transmission scenarios [33]. On the contrary, a study in a neighboring Pwani region reported a slightly higher prevalence in males [34]. This was similar to that reported by the Demographic and Health Survey and Malaria Indicator Survey [10] and other studies in north-east Tanzania [35] and Ethiopia [36]. The observed difference might be contributed by study population characteristics that also included primary healthcare facilities, geo-ecological differences, high exposure behaviour among males and other cultural factors that have been associated with malaria risk [37].

Wards with rice fields and other anthropogenic activities like bricks making had high prevalence [29, 38] and had previously been linked with higher entomological inoculation rates and malaria burden reflecting the presence of suitable microhabitats for malaria vectors [39]. The observed distribution might be coincidental as the study design was not powered to capture spatial distribution.

Despite the general decline of malaria cases in endemic areas including Tanzania, pockets of hotspots remain. Such hotspots are likely maintained by the high parasite density in a section of the population like what we observed in this study. Our findings call for a focused intensification of the preventive and control measures targeting high transmission areas to sustain the gains on malaria infections reductions.

## Limitations

The prevalence of malaria in this study could be under- or over-estimated because this study is a hospital-based survey and might not reflect the situation in the primary healthcare facilities and the population at large, however, these findings still provide important information on malaria at fine geographical scale.

## Data Availability

The datasets used and/or analysed during the current study are available from the corresponding author on reasonable request.

## Abbreviations

GMPD: Geometric mean parasite density
OPD: Outpatient department
WBC: White blood cells
WHO: World Health Organization
